# Precocious puberty in Korean girls with and without exposure to endocrine-disrupting chemicals in toy slime: a comparative analysis

**DOI:** 10.1186/s12902-021-00855-x

**Published:** 2021-09-18

**Authors:** Mi Seon Lee, Gi Min Lee, Cheol Woo Ko, Jung Eun Moon

**Affiliations:** 1 Department of Pediatrics, School of Medicine, Kyungpook National University, Kyungpook National University Hospital, Daegu, Republic of Korea; 2 Department of Pediatric Endocrinology, Kyungpook National University Children’s Hospital, 807, Hoguk-ro, Buk-gu, 41404 Daegu, Republic of Korea

**Keywords:** Endocrine disruptors, GnRHa treatment, Precocious puberty, Sex hormones

## Abstract

**Background:**

Toy slime is popular in Korea, and in parallel, pre-pubertal girls visit hospitals for early pubertal signs. Thus far, numerous studies have investigated the association of endocrine-disrupting chemicals (EDCs) with precocious puberty (PP). However, there is a lack of studies on the clinical manifestations and sex hormones. We aimed to investigate early pubertal development in Korean girls with or without a history of toy slime exposure and determine changes in bone age, Tanner stage, and sex hormones.

**Methods:**

In this study, 140 girls underwent stimulation tests at Kyungpook National University Children’s Hospital Endocrinology Department, during January 2018 and December 2020. Patients were divided into two groups for gonadotropin-releasing hormone (GnRH) stimulation test and frequency of exposure to toy slime (EDCs). GnRH stimulation test was conducted after an intravenous injection of 100 µg of luteinizing hormone-releasing hormone. Slime exposure was defined as *Slime* ≥ 3 times/week for ≥ 3 months.

**Results:**

History of slime exposure was found in 14 of 58 and 65 of 82 patients in the central PP (CPP) and non-CPP groups, respectively. Slime-exposed patients had advanced bone age, although their Tanner stage was low. Patients with a history of toy slime exposure were 5.5 times more likely to be diagnosed with non-CPP than patients without slime exposure (*p < 0.05*).

**Conclusions:**

Exposure to toy slime in prepubertal girls may be associated with rapid clinical advancement of pubertal development and bone age, and the patients appear more likely to be diagnosed with non-CPP.

## Background

Precocious puberty (PP) in girls is defined as a condition wherein the first pubertal sign occurs before the age of 8 years [1]. It is categorized into central PP (CPP), peripheral PP (PPP), and benign pubertal variants. In the gonadotropin-releasing hormone (GnRH) stimulation test, CPP is GnRH-dependent, while PPP is GnRH-independent [2]. The causes of PP vary, including idiopathic, central nervous system tumors, and ovarian tumors [3, 4]. Recently, the presence of endocrine-disrupting chemicals (EDCs) in the environment has received much attention as a factor that disturbs the onset and progression of pubertal development [5]. EDCs are found in many products, ranging from plastic products to metal food cans, cosmetics, and toys. The EDCs associated with PP are bisphenols (BPA), phthalates, and pyrethroids. Most studies on EDCs and PP conducted thus far have focused on analyzing the detected levels of EDCs [6]. In addition, a greater number of studies have investigated the detected levels and specific types of EDCs in patients previously diagnosed with CPP and compared the data with those in control subjects [7–10]. Thus, there is a lack of studies on EDC exposure frequency in patients with PP, its clinical manifestations, and effects on hormone test data. Since the past few years in Korea, a toy known as “toy Slime” has become highly popular. The Korean Agency for Technology and Standards of the Ministry of Trade, Industry, and Energy conducted an investigation of harmful substances present in the commercially available toy *Slime* in Korea and reported that the level of DEHP (di-2-ethylhexyl phthalate) in several slime toys exceeded the safety limit [11]. This study therefore investigated the history of toy slime exposure (history of exposure to EDCs) in patients who visited the hospital due to early pubertal development. This study also determined the changes in bone age (BA), Tanner stage, and sex hormones including GnRH stimulation test results.

## Methods

### Ethical approval

This study was approved by the Institutional Review Board of Kyungpook National University Chilgok Hospital, Daegu, Korea (approval number: 2020-04-029). As this was a retrospective study, the need for obtaining informed consent from patients was waived by the Institutional Review Board.

### Subjects

In this retrospective comparative analysis, 140 girls who underwent GnRH stimulation tests at Kyungpook National University Children’s Hospital Endocrinology Department due to PP between January 2018 and December 2020 were included. Using the classic definition, PP was defined as the first pubertal sign that occurs before the age of 8 years in girls [12]. GnRH stimulation tests were performed in all the patients. For analyses, study patients were divided into groups according to the GnRH stimulation test results and history of exposure to toy *Slime* (EDCs). The GnRH stimulation test was conducted between 09:00 and 10:00. After administering an intravenous injection of 100 µg of luteinizing hormone (LH)-releasing hormone (LHRH; Gonadorelin), blood samples were collected at 0, 15, 30, 45, and 60 min. Peak LH concentration of ≥ 5 mIU/L was defined as being representative of the pubertal pattern [13, 14]. History of exposure to slime was defined as exposure to *Slime* ≥ 3 times/week for ≥ 3 months at the time of GnRH stimulation tests. Age, BA, BA chronological age (CA), body mass index (BMI; z-score), Tanner stage, basal LH level, follicle-stimulating hormone (FSH) level, DHEA-s level, and GnRH stimulation test results were retrospectively analyzed for each subject. BA was measured by two experts using the method of Pyle and Glenlich, and the mean value was obtained [15]. Pubertal development was evaluated by two endocrinologists based on Tanner’s criteria.

### Statistical analyses

Statistical analyses were performed using IBM SPSS, version 23.0 (IBM Co., Armonk, NY, USA). The two-sample t-test was used to compare baseline characteristics between the two groups, and the chi-square test was used to evaluate the association between the history of exposure to slime (EDCs) and GnRH stimulation test results. Statistical significance was set at *p* < 0.05.

## Results

The mean age of the 140 prepubertal girls who visited the hospital for PP was 7.04 ± 0.98 years. The mean Tanner stage was 3.03 ± 0.68, and the mean BMI was 18.19 ± 2.99 kg/m^2^ (BMI for age z-score was 1.01 ± 1.50).

In total, 140 patients were categorized depending on the GnRH stimulation test results for a comparative analysis: 58 patients (41.4 %) were included in the GnRH (+) group and 82 patients (58.6 %) in the GnRH (–) group. The two groups showed no significant differences in CA or BA-CA and height SDS. BMI was significantly lower in the GnRH (–) group. The Tanner stage was significantly higher in the GnRH (+) group than in the GnRH (–) group (3.33 ± 0.75 vs. 2.83 ± 0.56, *P* < 0.05). Although the two groups showed no significant difference in basal LH levels, basal FSH and the peak LH/FSH ratio were significantly higher in the GnRH (+) group. A history of slime exposure was significantly more common (79.2 %) in the GnRH (–) group than in the other group (*P* < 0.05) (Table 1).

**Table 1:** Clinical and laboratory characteristics of the study groups based on GnRH stimulation test results

Characteristic	GnRH(+) (*n* = 58)	GnRH(-) (*n* = 82)	*p* value
CA(yr)	6.99 ± 0.82	7.08 ± 1.09	0.600
BA-CA(yr)	0.55 ± 1.22	0.95 ± 1.12	0.053
Tanner stage	3.33 ± 0.75	2.83 ± 0.56	< 0.05*
Height SDS	0.90 ± 1.54	1.28 ± 1.34	0.120
BMI z-score	0.50 ± 1.24	1.37 ± 1.58	< 0.05*
Basal LH(mIU/mL)	0.80 ± 3.47	0.08 ± 0.10	0.065
Peak LH(mIU/mL)	9.63 ± 9.25	2.38 ± 1.17	< 0.05*
Basal FSH(mIU/mL)	3.02 ± 1.60	1.77 ± 1.25	< 0.05*
Peak FSH(mIU/mL)	21.89 ± 7.69	14.32 ± 6.75	< 0.05*
Peak LH/Peak FSH	0.50 ± 0.48	0.19 ± 0.11	< 0.05*
Slime (EDCs) exposure(N, %)	14 (24.1 %)	65 (79.2 %)	< 0.05*

*GNRH* gonadotropin-releasing hormone, *CA* chronological age, *yr* years, *BA* Bone age, *BMI* body mass index, *LH* Luteinizing hormone, *FSH* Follicle-stimulating hormone, *N* number, *EDCs *endocrine-disrupting chemicals

^*^P value <0.05

**Fig. 1:**
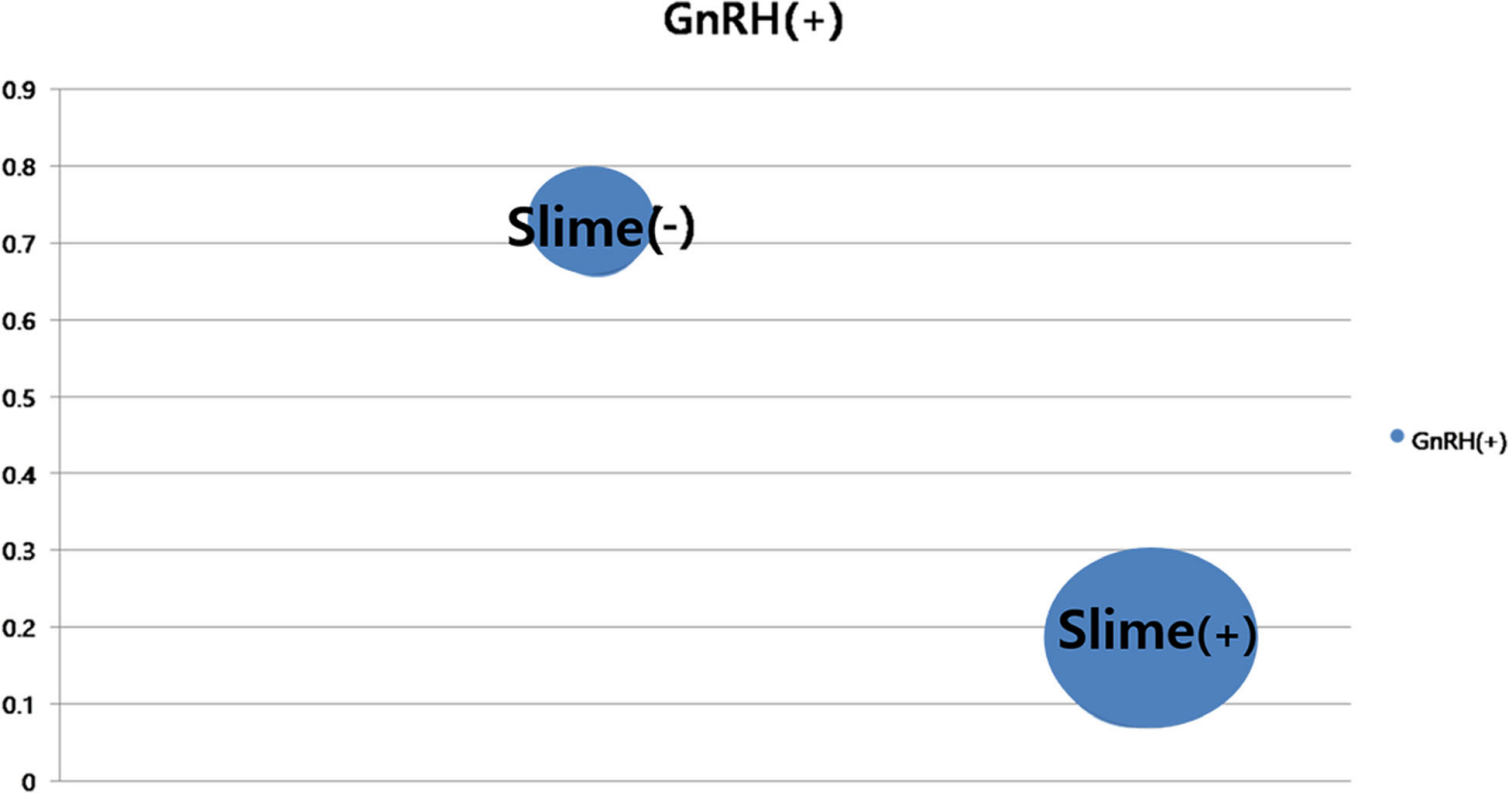
Association between history of exposure to toy slime (EDCs) and GnRH stimulation test results. The possibility of a diagnosis of non-CPP was 5.55 times higher in patients with toy Slime exposure than in those without the exposure (*P *< 0.001). Results of the chi-square test for the comparison between the two groups. EDC, endocrine-disrupting chemical; GnRH, gonadotropin-releasing hormone

A total of 140 patients were categorized depending on their toy slime (EDC) exposure history for a comparative analysis: 79 patients were included in the Slime (+) group and 61 patients in the Slime (–) group. BA-CA was significantly higher in the Slime (+) group than in the other group (0.92 ± 1.3 vs. 0.59 ± 1.15, *P* < 0.05). Height SDS was significantly higher in the Slime (+) group than in the other group (1.37 ± 1.29 vs. 0.80 ± 1.55, *P* < 0.05). The Tanner stage was slightly lower (2.92 ± 0.64 vs. 3.18 ± 0.74, *p* < 0.05) in the Slime (+) group than in the other group. BMI showed no significant intergroup differences. There was no significant difference in the basal LH level between the two groups, whereas basal FSH and the peak LH/FSH ratio were significantly lower in the Slime (–) group than in the other group. The number of patients with a positive result in the GnRH stimulation test was 14 (17.7 %) in the Slime (+) group and 44 (72.1 %) in the Slime (–) group, indicating that it was more frequent in the Slime (+) group than in the Slime (–) group (Table 2). The results of the chi-square test that compared the Slime (–) and (+) groups predicted that the likelihood of a diagnosis of non-CPP was 5.55 times higher in patients with toy Slime exposure than in those without the exposure (*P* < 0.001) (Fig. 1).

**Table 2:** Differences in clinical and laboratory characteristics according to toy slime (EDC) exposure

Characteristic	Slime (+) (*n* = 79)	Slime (-) (= 61)	*p* value
BA-CA(yr)	0.92 ± 1.30	0.59 ± 1.15	< 0.05*
Tanner stage	2.92 ± 0.64	3.18 ± 0.74	< 0.05*
Height SDS	1.37 ± 1.29	0.80 ± 1.55	< 0.05*
BMI z-score	1.18 ± 1.54	0.79 ± 1.45	0.131
Basal LH(mIU/mL)	0.43 ± 2.90	0.31 ± 0.89	0.733
Peak LH (mIU/mL)	3.70 ± 5.23	7.80 ± 8.17	< 0.05*
Basal FSH(mIU/mL)	1.96 ± 1.44	2.71 ± 1.54	< 0.05*
Peak FSH(mIU/mL)	15.79 ± 7.27	20.06 ± 8.47	< 0.05*
Peak LH/Peak FSH	0.25 ± 0.26	0.42 ± 0.43	< 0.05*
GnRH positive(N, %)	14 (17.7 %)	44 (72.1 %)	< 0.05*

*EDCs *endocrine-disrupting chemicals, *CA* chronological age, *yr* years, *BA* Bone age, *BMI* body mass index, *LH* Luteinizing hormone, *FSH* Follicle-stimulating hormone, *N* number, *GNRH* gonadotropin-releasing hormone, *GnRH(*+) Percentage of positive of GnRH stimulation test

^*^P value <0.05

Among the 140 patients, 82 patients with a negative result in the GnRH stimulation test (non-CPP patients) were divided according to their toy slime exposure history for a comparative analysis. The number of non-CPP patients in the Slime (+) group was 65/82 (79.2 %), whereas the number of non-CPP patients in the Slime (–) group was 17/82 (20.8 %). CA, BA-CA, Height SDS, and Tanner stage showed no significant intergroup differences. The basal LH and peak LH levels showed no significant difference, while the peak LH/FSH ratio was significantly lower in the Slime (+) group than in the other group (0.17 ± 0.11 vs. 0.24 ± 0.13, *p* < 0.05) (Table 3).

**Table 3:** Differences in clinical and laboratory characteristics in the non-CPP group according to toy slime exposure

Characteristic	Slime(+) (*n* = 65)	Slime(-) (= 17)	*p* value
CA(yr)	7.03 ± 1.02	7.27 ± 1.31	0.433
BA-CA(yr)	0.96 ± 1.13	0.62 ± 1.02	0.285
Tanner stage	2.83 ± 0.60	2.82 ± 0.39	0.963
Height SDS	1.41 ± 1.25	0.79 ± 1.58	0.092
BMI z-score	1.64 ± 2.06	1.89 ± 1.86	0.624
Basal LH(mIU/mL)	0.087 ± 0.01	0.070 ± 0.002	0.569
Peak LH (mIU/mL)	2.26 ± 1.11	2.83 ± 1.29	0.073
Basal FSH(mIU/mL)	1.83 ± 1.32	1.55 ± 0.92	0.320
Peak FSH(mIU/mL)	14.59 ± 9.73	13.28 ± 6.94	0.721
Peak LH/Peak FSH	0.17 ± 0.11	0.24 ± 0.13	0.027*

*CA* chronological age, *yr* years, *BA* Bone age, *BMI* body mass index, *LH* Luteinizing hormone, *FSH* Follicle-stimulating hormone, *N* number

^*^P value <0.05

## Discussion

This study reports the results of a comparative analysis of clinical and GnRH stimulation test results based on the toy Slime (EDCs) exposure history of patients visiting the hospital due to PP. The frequency of non-CPP diagnosis was higher in the Slime (+) group that showed advanced BA-CA. Moreover, the Tanner stage was lower in the Slime (+) group, with a significantly lower peak LH/FSH in the GnRH stimulation test.

Previous studies regarding exposure to EDCs, including phthalate and PP, have reported controversial results. The study by Chou in 2009 [7] and that by Wolff in 2010 [16] reported a high frequency of phthalate detection in CPP patients, whereas the study by Buttke in 2012 and that by Frederiken in 2012 reported no significant difference [17, 18]. As such, the results of studies regarding EDC and PP have been controversial. In this study, patients with pubertal signs and advanced BA were examined; patients with a negative result in the GnRH stimulation test accounted for 82 out of 140 (58.5 %), indicating that a high proportion of those with PP had non-CPP. For the 82 patients diagnosed with non-CPP, a slime (EDC) exposure history was found in 79.2 % of patients. In an analysis based on lime exposure history, the frequency of CPP diagnosis in patients with a history of slime exposure among those visiting the hospital due to PP was relatively low at 17.7 %, while the frequency of CPP diagnosis in patients without a slime exposure history was high at 72.1 %. Thus, a patient with a history of slime exposure is more likely to be diagnosed with non-CPP rather than with CPP, which suggests that a slime exposure history may lead to non-CPP.

BA advancement is commonly observed in patients with PP. It is well known that sex hormones are closely associated with the maturation of the epiphyseal plate [19]. In the study by Buluş et al., the EDC detection frequency was high in CPP patients, with a significantly high level of bone advancement. In this study, the frequency of non-CPP diagnosis was high in patients with a history of slime exposure, who simultaneously showed an advancement of BA [20]. It is presumed that exposure to EDCs could have promoted the advancement of BA, as previously reported. Buluş et al. reported no change in basal hormone levels when phthalate detection was performed in patients with CPP and PPP [20]. Similarly, in this study, no notable intergroup difference was found in the basal hormone levels. However, in patients in the GnRH (–) group with a history of slime exposure, the peak LH/FSH ratio in the GnRH stimulation test was significantly lower. It is well known that EDCs influence ovarian folliculogenesis and ovarian function in adults [21]. Hannon et al. reported that phthalate exposure in mice accelerates primordial follicle recruitment [22, 23]. This suggests that EDC exposure in prepubertal girls further promoted the advancement of BA, which may be due to the activation of FSH rather than LH, leading to the progression to non-CPP. Thus, the findings of this study indicate a high probability of the development of non-CPP in patients with a history of slime exposure, and the possibility of PP induced by folliculogenesis due to FSH activation. In the future, additional studies should be conducted regarding the disturbance and association between slime and folliculogenesis in pediatric patients.

Furthermore, from a clinical perspective, the association between exposure to slime and PP is clear; however, the challenge lies in screening for indications that require GnRH agonist treatment. In this study, the correlation between slime exposure and GnRH stimulation test results was determined using the chi-square test. The probability of a patient with a history of Slime exposure diagnosed with CPP was 5.55 times lower, a statistically significant difference. This indicates a high probability of a negative result in the GnRH stimulation test for patients with a slime exposure history when they visit the hospital due to PP. In addition, while BA advancement and Height SDS were significantly higher in patients with a slime exposure history, the Tanner stage was low, and there was no difference in BMI.

The investigation in this study focused on the history of exposure to toy slime (EDC exposure history), while the limitation is that the specific type of EDC in each slime product and the detected amount of EDC were not analyzed. Further studies should examine the EDCs detected in the urine and blood of patients with a slime exposure history and the EDCs detected in the slime product itself.

## Conclusions

Exposure to toy slime in prepubertal girls may be associated with rapid clinical advancement of pubertal development and bone age, and the patients appear more likely to be diagnosed with non-CPP.

## Data Availability

The datasets used and analyzed during the current study are available from the corresponding author upon reasonable request.
